# High-precision spatial localization of mouse vocalizations during social interaction

**DOI:** 10.1038/s41598-017-02954-z

**Published:** 2017-06-07

**Authors:** Jesse J. Heckman, Rémi Proville, Gert J. Heckman, Alireza Azarfar, Tansu Celikel, Bernhard Englitz

**Affiliations:** 1 Department of Neurophysiology, Donders Institute for Brain, Cognition and Behaviour, Radboud University, Nijmegen, The Netherlands; 2 Department of Mathematics, Institute for Mathematics, Astrophysics and Particle Physics, Radboud University, Nijmegen, The Netherlands

## Abstract

Mice display a wide repertoire of vocalizations that varies with age, sex, and context. Especially during courtship, mice emit ultrasonic vocalizations (USVs) of high complexity, whose detailed structure is poorly understood. As animals of both sexes vocalize, the study of social vocalizations requires attributing single USVs to individuals. The state-of-the-art in sound localization for USVs allows spatial localization at centimeter resolution, however, animals interact at closer ranges, involving tactile, snout-snout exploration. Hence, improved algorithms are required to reliably assign USVs. We develop multiple solutions to USV localization, and derive an analytical solution for arbitrary vertical microphone positions. The algorithms are compared on wideband acoustic noise and single mouse vocalizations, and applied to social interactions with optically tracked mouse positions. A novel, (frequency) envelope weighted generalised cross-correlation outperforms classical cross-correlation techniques. It achieves a median error of ~1.4 mm for noise and ~4–8.5 mm for vocalizations. Using this algorithms in combination with a level criterion, we can improve the assignment for interacting mice. We report significant differences in mean USV properties between CBA mice of different sexes during social interaction. Hence, the improved USV attribution to individuals lays the basis for a deeper understanding of social vocalizations, in particular sequences of USVs.

## Introduction

Mice emit complex and non-random ultrasonic vocalizations (USV) during social interactions^1–4^. These vocalizations are strongly modulated on different levels by a variety of contextual determinants, such as age, genetic background, behavioural state and to a lesser extent by sex^5^. Mice utilize these USVs during social interactions and in distress to mediate essential behaviours^6–12^. Recently, it has been shown that playback of different types of USVs can also elicit different responses in the recipient mouse^13^. This suggests that mice are able to adapt their vocal behaviour in a context-dependent manner and that these modifications may convey information^14^. One of the most commonly used experimental paradigms to study mouse USVs is the dyadic social interaction^6, 8, 15–23^, i.e. the interaction of a pair of mice during behaviors such as courtship^24–31^ and territorial disputes^8^.

By its very nature, vocal behaviour is thus highly susceptible to contextual modulation. Hence, in social interactions, one of the main experimental challenges is to attribute USVs properly to their emitter, e.g. an adult male mouse’s courtship call. However, as mice do not show clear visual cues of their vocal behaviour in these interactions^7^, this attribution has been difficult. Often it has simply been assumed which animal vocalised. For instance during courtship, vocalizations are primarily attributed to the male mouse^29, 32^. Control experiments involving devocalised males, indeed suggest that the female mouse does not vocalize during these specific events^18, 32^. However, this does not completely exclude female vocalizations during male-female interactions altogether. For instance, female mice may still vocalize but only in response to male calls. Thus, due to the indistinctness of USV origin in the literature it is not certain, whether some putatively defined male courtship calls, may have been female instead. Social interaction paradigms with male-male pairings may rule out the presence of female USVs^8^, but these calls can no longer be compared to male courtship calls, due to changes in behavioral state.

A recent study shows that female mice in fact do vocalize during social interaction with males, specifically in between the chases of courtship behaviour^33^. For this purpose, a microphone array was used in addition to a sound source localization method^34^. In total, 18% of the USVs here were assigned to female mice. Most of these USVs occurred within 1 second of a male USV, suggesting an exchange of information during this event. However, the median error between the estimated source and actual source of USV location was 38.7 mm, which leaves some room for erroneous assignments and may not be sufficient in other paradigms, such as social facial interaction^35–37^.

Presently, we devise and evaluate a more accurate estimation technique for localizing and assigning USVs to animals that takes both temporal and level differences into account. The present method is able to reduce the source localization error substantially, depending on the stimulus class (about a factor of 4 for USVs). While the present results are obtained on a 1D setup using two microphones, the methods presented can in principle be generalised to 2D or 3D contexts by adding microphones and slightly generalizing the analytical correction method.

## Results

**Figure 1:**
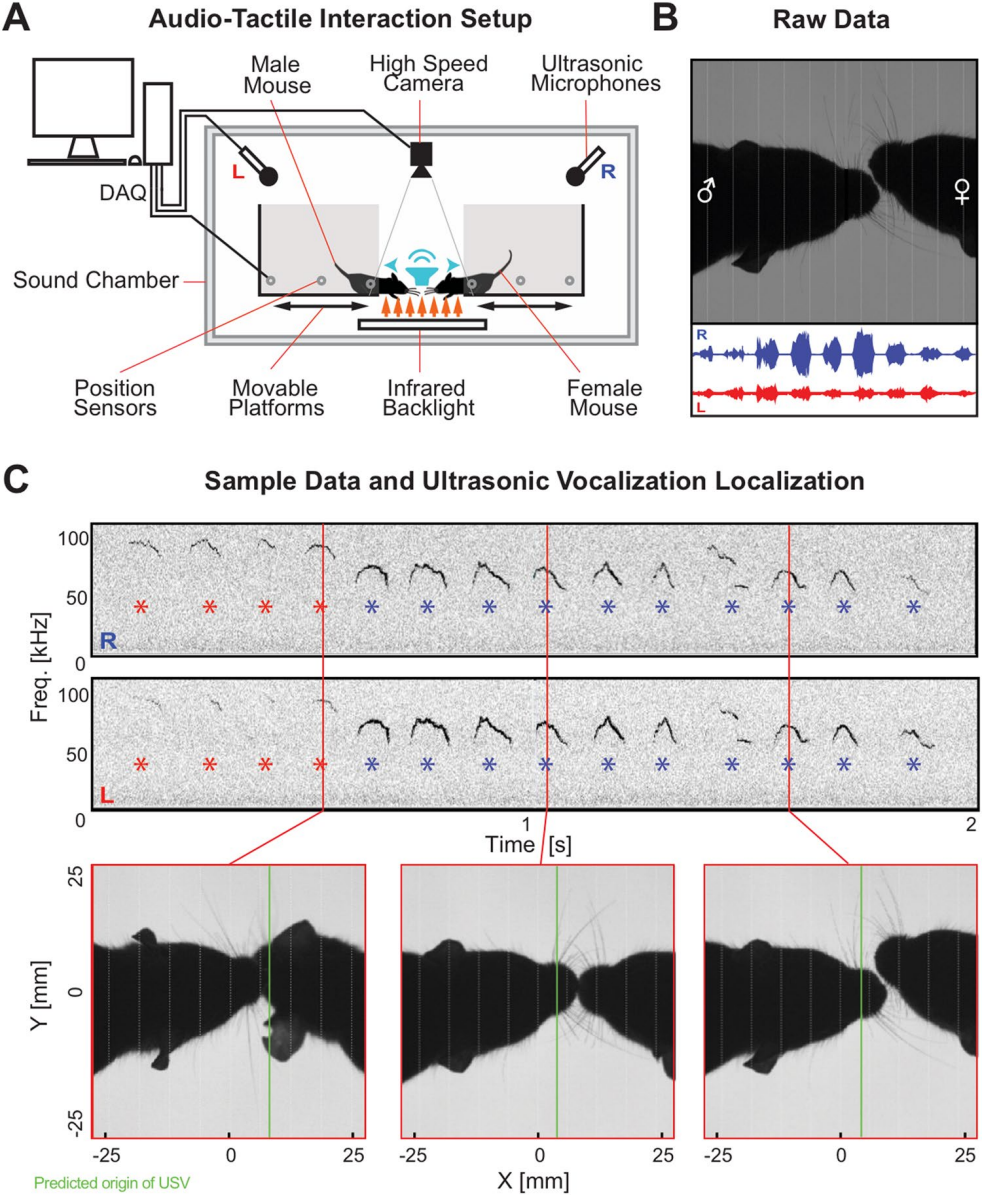
The study of mouse vocalizations during natural behavior requires attributing individual vocalizations to individual mice. (**A**) For development and testing of localization algorithms a dedicated interaction space was designed. Mouse vocalizations were recorded during social interactions of a male-female pair of CBA mice. Mice were located on separated platforms, which allowed them to interact by making snout contact, but not cross the platforms. The entire setup was housed in a sound attenuated chamber. For testing and calibration of the algorithms, localization performance was in addition assessed using a movable speaker, which was positioned at a set of locations that could reflect mouse positions (indicated by the blue speaker). For detailed spatial dimensions of the setup, see Methods. (**B**) Positions were estimated based on high-speed video-images captured from directly above (upper part), and also based on mouse vocalizations recorded at two locations above the platforms (lower part; channels are represented in blue and red). (**C**) Mice vocalised primarily during social interaction, where they are in close proximity. Attribution of vocalizations is complicated by the partial overlap of the snouts.

We developed and experimentally evaluated a range of sound source localization algorithms in the context of mouse vocalizations, especially during social interactions. Experimental data from 6 male and 4 female mice comprising a total of ~4000 vocalizations entered the analysis. Animals interacted *ad libitum* with their snouts on elevated platforms, separated by a gap, while both high-speed video and two audio signals were collected (Fig. 1A,B). Mouse USVs occurred primarily during and after social interaction, where the attribution of a USV to a mouse is challenging (Fig. 1C). The accurate estimation of sound source location involves two steps: First, estimate the inter-microphone delay (IMD), and second, compute the translation of the IMD to a position in space, based on the geometry of the setup. To further introduce the experimental recording condition, we proceed directly by describing the second step in the following section.

### Analytical compensation for vertical source-microphone distance

**Figure 2:**
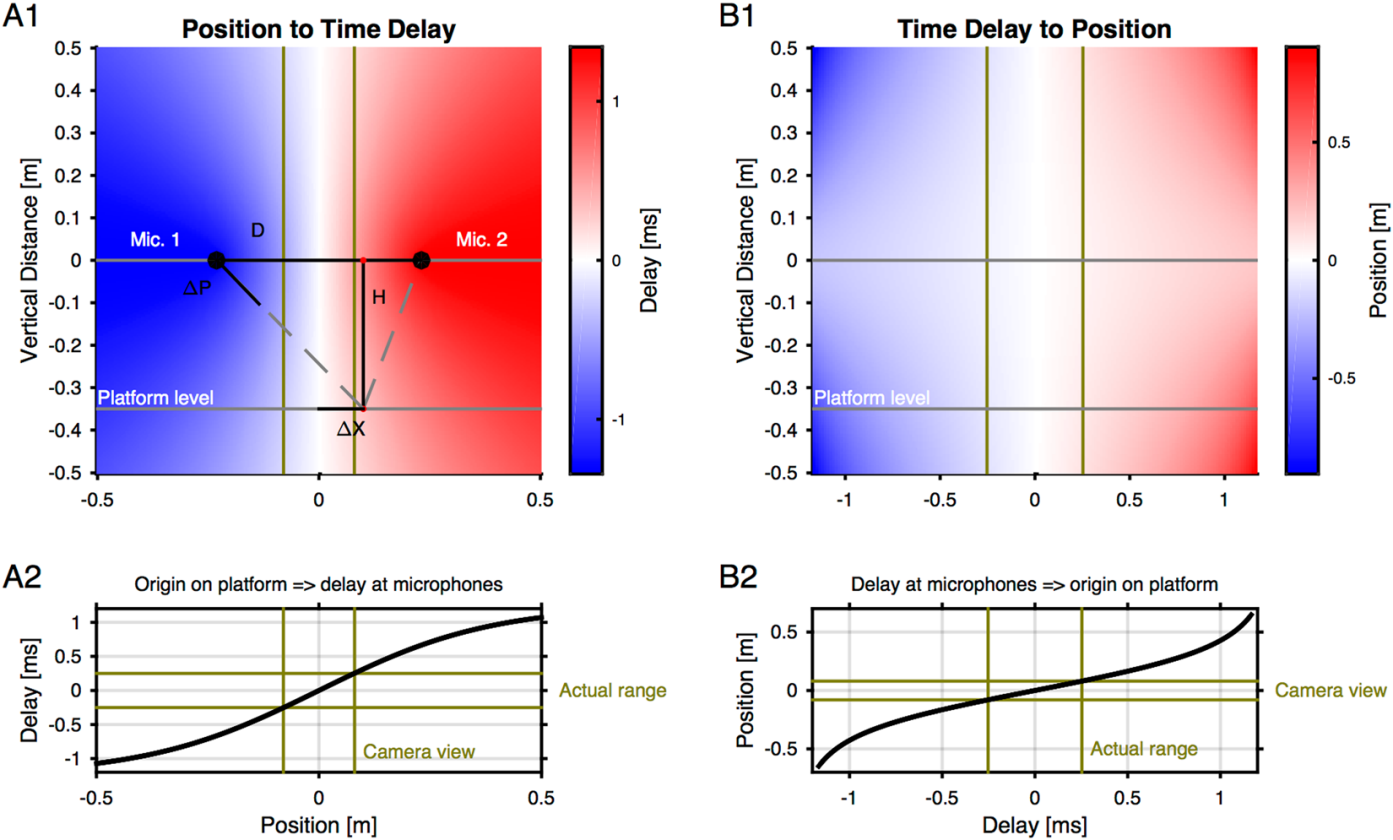
Analytical solution to account for the dependence of inter-microphone delay on microphone elevation. (**A1**) The position of a sound source relative to the microphones determines the difference in their sound arrival times (negative values indicate arrival at Mic. 1 before Mic. 2, computed using Eq. 6. The variables are indicated to visualize the computation. *ΔP* = Path difference from sound source to the two microphones; *ΔX* = Position sound source relative to the center; H = Microphone height relative to the platform; D = Distance between the microphones). Since the microphones are typically not positioned at the same height as the animal snouts, this difference in arrival times depends on the horizontal position and the relative height between snouts and microphones (colour coded here for all potential sound source positions). (**A2**) As a function of horizontal position, the dependence is sigmoidal, centered between the microphones and a slope which depends on the microphone height. Depicted here is the position-to-time relation for the dimensions of the present setup (microphone height: 356 mm). The inter-microphone delay ranges roughly between [−1,1] ms, i.e. less than maximally possible ([−1.25,1.25] ms), if the microphones were on the same height as the platform. In the camera’s view field (between vertical green), the dependence is close to linear. This leads to the relevant range of delays which can be compared to camera positions. (**B1**) To obtain the sound source position from the inter-microphone delay, the position-delay relationship has to be inverted. We computed this analytically (see Eq. 12), which leads to hyperbolic-type shapes for a given platform level. (**B2**) For the present setup, the position as a function of inter-microphone delay is relatively flat, and very close to linear in the region of the camera view. The formulas allow a computation of the horizontal position from the inter-microphone measurement.

The signals at the two microphones can be used to compute an inter-microphone delay (IMD) for each vocalization. Converting the IMD *ΔT* via the speed of sound to a distance can be used to estimate the sound source location. The plain value corresponds to the difference in distance *ΔP* of the sound source to the microphones *only if* the microphones are at the same height as the sound source (see Fig. 2A for illustration). In all other cases, *ΔP* will be smaller. The magnitude of the effect depends on the relative vertical distance between the sound source and the microphones. We have derived an analytical relationship for determining the lateral position *ΔX* from the IMD *ΔT* (Eq. 12), and vice versa (Eq. 6).

The IMD can be computed directly from *ΔX* on the basis of the geometry (see Methods, Eq. 6, and Fig. 2A). The shape of the dependence is generally sigmoidal as a function of *ΔX*, with its steepness decreasing as a function of vertical distance H, i.e. larger *ΔX* lead to less change in *ΔT*, thus complicating the task. Inter-microphone distance was not varied in the depiction, but would increase the maximal IMD and thus the resolvable range of positions. For the present spatial arrangement (H = 356 mm, D = 460 mm) the overall range of delays is +/−1.25 ms, however, the range covered by the high-speed camera is maximally 160 mm (2nd set of experiments), thus corresponding to a range of delays of only +/−0.25 ms (Fig. 2B1). Within the camera range, a linear approximation would be quite accurate, although the analytical form was used here.

The inverse relationship, computing *ΔX* from *ΔT* is less trivial, but can still be solved analytically (see Methods, Eq. 12 and its derivation). The shape is generally similar to a tangent function, with acceleration towards the limits of the delay range (as above: +/−1.25 ms). The greater the vertical distance between source and microphones, the steeper the dependence of *ΔX* on *ΔT* (Fig. 2A1), meaning that small errors in delay estimation lead to larger errors in position estimation. For the present configuration the range of camera positions can be reestimated from the available delays (Fig. 2B2).

The analytical correction for the vertical distance between microphones and the source is important to avoid systematic errors in estimation. The same technique should be applicable for localization in 2D and 3D cases (see Discussion).

### Ground truth comparison based on artificial stimuli

**Figure 3:**
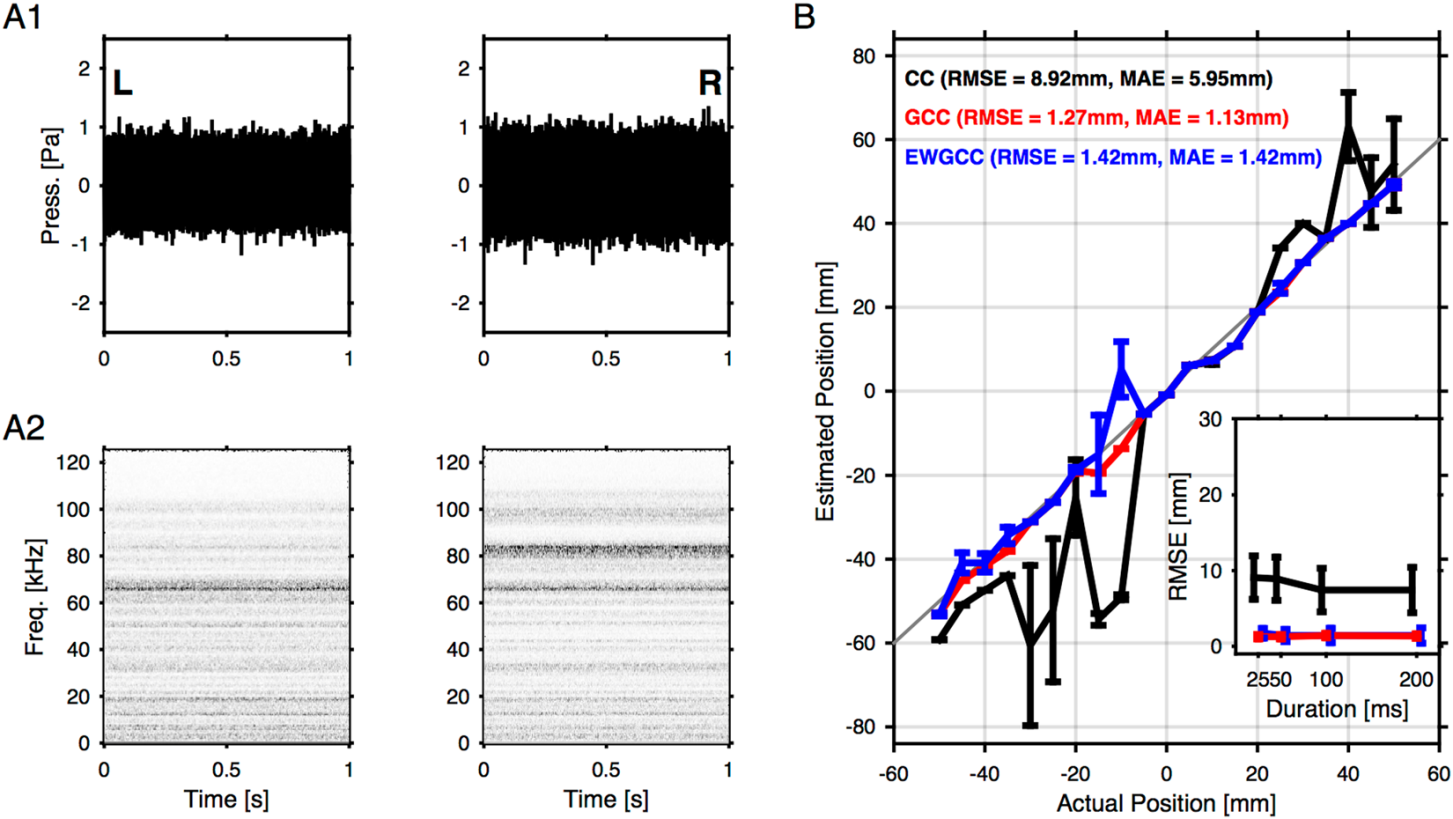
Ground truth comparison for artificial broadband sounds. The accuracy of the different estimation algorithms was compared on the basis of artificial sounds (Gaussian noise), which were presented using a movable speaker at a range of locations, i.e. −50 mm to 50 mm at 5 mm steps (see Figs 1 and S2 for setup/speaker details). (**A**) The recorded sound at the left (A1, left) and the right (A1, right) microphone were similar in level, but differed in frequency content (compare spectrograms in A2 left with A2 right). Since the speaker was well equalised (see Fig. S1), these spectral differences must stem from reflections inside the apparatus In addition, they also depended on the speaker location (not shown). (**B**) The localization methods were evaluated across the entire range. The generalised cross correlation (GCC, red) method performed best, with a median residual RMSE = 1.27 mm (MAE = 1.13 mm). In comparison, the basic cross-correlation (CC, black) diverged erratically outside the central range of positions, leading to a substantially greater median RMSE = 8.92 mm mm (MAE = 5.95 mm). The envelope weighted generalised crosscorrelation (EWGCC, blue) performed almost as well as GCC with an RMSE = 1.42 mm (MAE = 1.42 mm). Results show averages over 10 random draws, and error bars represent 1 SEM. The quality of localization showed only a slight dependence on available data, reaching precise localization already for segments of 25 ms duration (inset). The errorbars in the inset show [14,86]% percentiles, i.e. indicate the level of variability of these estimates.

We compared and validated the estimation methods using a set of acoustic stimuli presented across a range of relevant locations during social interaction ([−50, 50] mm, in steps of 5 mm). The stimuli were simple Gaussian white noise bursts (see Fig. 3A), intended to provide an upper bound to the accuracy of the estimation methods. The wide frequency range of this stimulus avoids ambiguities and should constitute an easy challenge for localization algorithms. Stimuli were presented via a calibrated audio system which covered a frequency range encompassing the range of presented stimuli ([10–100] kHz, see Fig. S2 and Methods). The speaker was placed at a fixed height and variable positions mimicking those of the mice in the same booth (see Fig. 1A). The Gaussian white noise was always played for 1 s, but sound localization performed on subsections of different lengths (see Fig. 3B, inset).

Basic cross-correlation exhibited instability in several locations over the tested range (Fig. 3B, *CC*, black), leading to saltatory deviations by up to ~40 mm. These misestimates were systematic, i.e. repeated stimulation from the same position led to the same estimate, as indicated by the small error bars (SEM, computed from 10 repetitions of the identical stimulus from the same location). For basic CC we obtained an overall RMSE = 8.92 mm (MAE = 5.95 mm).

Generalised cross-correlation (GCC) estimates were robust and accurate, achieving a vastly improved RMSE = 1.27 mm (MAE = 1.13 mm, Fig. 3B, red), with essentially no difference in accuracy across the range of tested locations. For some locations the vanishing error bars indicate, nonetheless, that the estimation errors are not random (across repeated acoustic presentations of the stimulus).

Envelope weighted generalised cross correlation (EWGCC) performed similarly well as GCC with a MSE = 1.42 mm (MAE = 1.42 mm). The slight reduction in performance is caused by the exclusion of certain frequencies for estimation based on the envelope criterion, which means some - in this case relevant - information is ignored.

None of the three methods showed a strong dependence on sample size (see inset, colours corresponding to main panel), here evaluated at 4 different sample durations ranging from 25 to 200 ms.

GCC and EWGCC proved to be more reliable and precise than CC for sound localization of white noise. We also tested some additional methods (based on either phase, impulse response or spectrogram), which performed less reliably (see Discussion for more details).

### Ground truth comparison based on real vocalizations

**Figure 4:**
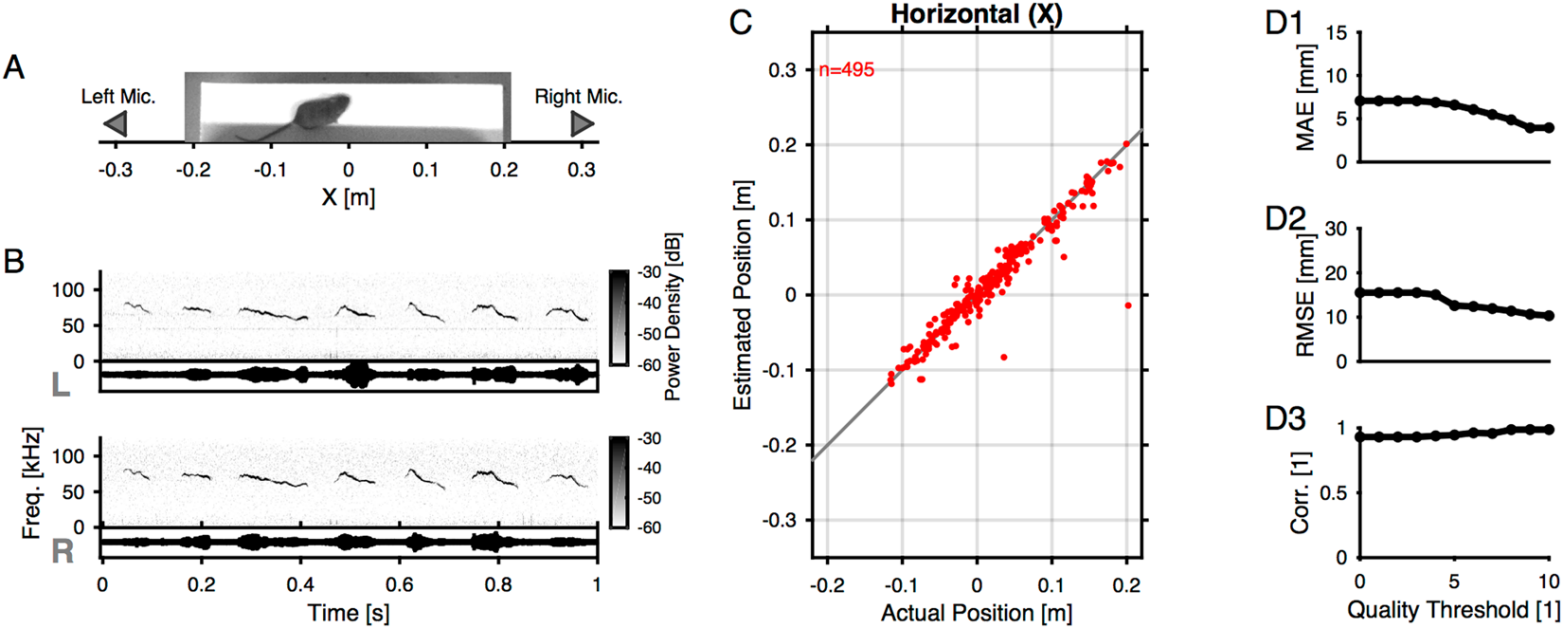
Localization of USVs from a single mouse. (**A**) Schematic of the recording setup and sample image. The mouse was free to move on the platform, and was repetitively brought into snout-snout contact with a female mouse ~30 s after the last vocalization. The female mouse was placed into a sound proofed box immediately after to primarily record male vocalizations. The platform was padded with acoustic foam and a very soft cloth to reduce movement noise. (**B**) The vocalizations emitted by the male mice under this condition resembled the vocalizations observed during social interaction in the gap interaction setup. The shapes of the USVs are well conserved across the two microphones (top: left, bottom: right), while the amplitude of the vocalizations differs naturally based on the direction of vocalization. (**C**) The actual vs. estimated positions corresponded well with each other (n = 3 mice). The depicted data is shown for correlation quality measures (CQM) > 6 (see Methods). The few outliers may be a due to environmental noise, as they also exhibited clear correlation peaks. (**D**) Estimation quality was assessed via the median average error (MAE, D1), the root mean squared error (RMSE, D2) and the correlation (Spearman rank correlation, D3). The precision of the estimates improved with CQM, where results are displayed as a function of quality threshold (i.e. for USVs with CQM greater or equal). MAE converged to ~4 mm, MSE to about 10 mm, and correlation reach values above 0.99.

**Figure 5:**
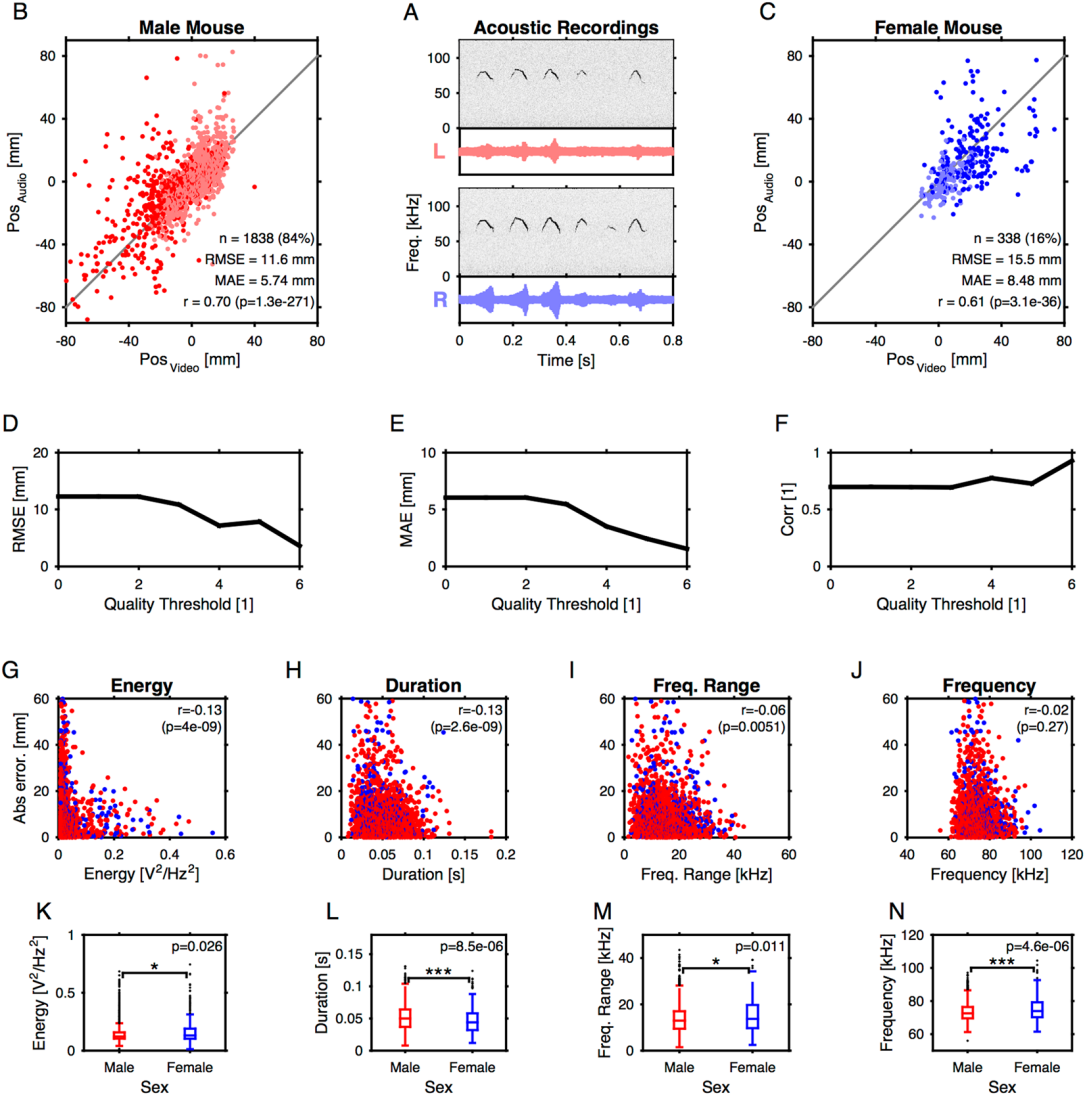
Properties of vocalizations during snout-snout interaction. (**A**) Acoustic recordings of mouse vocalizations were collected with two microphones (Top, left (red) microphone; bottom: right (blue) microphone). While the level of each vocalization varied between the microphones, the spectrotemporal structure remains well resolved in both. (**B**) The majority of the vocalizations were attributed to the male mouse (~84%), either based on location along (dark red) or based on a combination of position and relative level at the two microphones (light red, for cases where the mouse snouts were within 10 mm of each other). The actual location of the mouse head, estimated from the video (abscissa) was well predicted by the audio-based estimate (ordinate). (**C**) A smaller number of vocalizations was attributed to the female mouse (~16%). The estimated locations also agree well with the actual position of the female mouse exhibiting a solid correlation. (**D–F**) If vocalization were selected based on different CQM thresholds, the localization quality improved as measured by RMSE (**D**), MAE (**E**) and Spearman correlation (**F**). (**G–J**) The quality of localization dependent significantly on a USV’s energy (**G**), duration (**H**), and frequency range (**I**), however, was not significantly correlated with mean frequency itself (**J**). (**K–N**) The properties of vocalization differed between the sexes. Male calls had significantly lower mean frequency (Wilcoxon rank sum test, p ≪ 0.001; **J**) and longer durations (Wilcoxon rank sum test, p ≪ 0.001; **H**) than females calls, while energy (**G**) and frequency range (**I**) exhibited borderline significances (p < 0.05).

The localization methods were further verified under more realistic conditions, i.e. for vocalizations of a single mouse on an elongated platform (see Fig. 4A and Methods for details and dimensions). A female mouse was presented to the male mouse for snout-snout interaction, and then quickly removed and placed in a sound-attenuated box. Subsequent vocalizations were used for comparing estimated with actual positions, determined from the video. The male mouse emitted sequences of typical, inverted-u vocalizations (Fig. 4B), comparable to the ones emitted during the extended snout-snout interactions (see Fig. 5).

The estimated positions conformed with the actual positions over the entire range of locations along the platform (Fig. 4C, depicted are only vocalizations that fulfilled the CQM > 6 criterion, i.e. 495 out of a total of 897). The errors depended on the choice of the CQM threshold in a monotonic manner (Fig. 4D 1–2). The MAE reduced from 7 to ~3.95 mm for the highest chosen CQM (10), where the precision appears to reach a plateau. Similarly the RMSE decreased as a function of CQM, and reached a lower limit around 10 mm. Finally, the correlation between the estimated and the true positions rose from 0.97 to >0.99 for the highest CQM thresholds. We acknowledge that in the present paradigm it may be possible that a small number of female vocalizations were included in the male vocalization estimate, between the presentation of the female and the point when it was completely enclosed in the box. However, since this would introduce additional errors, this makes the current estimate an upper bound on the actual accuracy. The precision of both cross-correlation and generalised cross-correlation was also evaluated, but lead to significantly greater errors, MAE = 8.4 mm (RMSE = 17.4 mm) and MAE = 9.7 mm (RMSE = 18.9 mm), respectively (without CQM threshold). The relatively poor performance of GCC on vocalizations, as opposed to noise, derives from a tendency to localize towards the center, likely based on contributions from irrelevant frequencies, which was overcome by the band-limitation used in EWGCC. Therefore, only the EWGCC is evaluated during social interaction (see below).

In summary, the new EWGCC method in combination with the height correction approach (Eq. 12) and the CQM threshold leads to a high precision in the localization of USVs in 1D. The MAE of 4–7 mm in 1D compares favorably to the 1D localization error in Neunuebel *et al*.^33^ (estimated ~21.7 mm, converted from the 2D error of 38.7 mm via MAE of 1D and 2D Gaussian distributions). The difference in accuracy between artificial and real USVs was a surprise to us, and may be caused by the variable head-position of the animal, which may have influenced the effective path length to the speakers to some degree, while the orientation of the speaker was standardized.

### Application to social interaction of mice

Rodents and mice in particular vocalize in the ultrasonic range during social encounters^38, 39^. The role of individual animals during a social interaction has been difficult to assess, since it has been hard to assign individual vocalizations to an animal. We used the above described combination of empirical and analytical methods to perform this task in 1D. Vocalizations were recorded while a male-female pair of CBA mice interacted over a gap (see Methods, and Fig. 1 for details on the paradigm). Vocalizations were typically recorded at a signal-to-noise ratio of 2.4, as measured by computing the standard deviation of the sound pressure compared to the standard deviation of the noise, and can be easily discerned in the spectrogram of the left and right microphones (Fig. 5A, sequence of vocalizations, from left (top, red) and right (bottom, blue) microphones, estimated to come from the male mouse).

Generally, the snouts of the interacting animals can overlap, thus introducing ambiguity into the position estimate. Presently, this ambiguity is partly due to the use of only two microphones, allowing the localization to be performed in one dimension only. This problem can be addressed either by (i) determining the location in more directions, or by (ii) estimating the direction of vocalization. The constraints of the interaction setup made it impractical to introduce additional microphones, and we therefore used relative level at the two microphones to disambiguate the cases of overlapping snout positions. More precisely, if the animal snouts were within 2 times the MAE for 1D localization, i.e. 2 × ~5 mm = ~10 mm, we used the relative amplitude at the two microphones to determine the vocalizing animal, i.e. always the animal opposite to the microphone with the higher amplitude signal (see Fig. S3 for more details). We checked that the animals were actually facing in opposite directions, which is, however, the typical case for snout-snout interactions (see Fig. S3A). Further, we are confident that echoes inside the booth can be excluded as sources. Firstly, because most of the recordings were performed in platforms padded on the inside with acoustic foam with strong ultrasonic absorption properties. And secondly, because their localizations would fall far outside the central region. We visually separated the USVs attributed by position only (full colours) from the ones attributed by the combination of position and level (light colours, naturally occurring mostly in the middle) in Fig. 5.

The large majority of USVs were attributed to the male mouse (~84%, i.e. 1838/2176 Fig. 5B). The acoustically estimated position was significantly correlated with the male’s position estimated from the video recordings (r = 0.7, p < 10^−200^) with an RMSE = 11.6 mm (MAE = 5.74 mm, without selection by the CQM threshold). The remaining USVs were attributed to the female (~16%, i.e. 338/2176, Fig. 5C) with an RMSE = 15.5 mm (MAE = 8.48 mm). These numbers are consistent with an estimate of 18% for female C57Bl/6 J mice provided by previous work^33^.

As for the ground truth estimates (Fig. 4), the accuracy of the location estimate could be enhanced by selecting USVs based on different CQM thresholds. The average RMSE reduced from 12.1 mm to about 4 mm (at CQM > 6, Fig. 5D), the MAE reduced from 6 mm to ~2 mm (Fig. 5E), and the correlation coefficient increased from 0.7 to 0.95 (Fig. 5F).

### Dependence of estimation quality on general vocalization properties

The quality of spatial localization could exhibit some dependence on the spectrotemporal properties of the vocalization. USVs vary naturally in their level, length and frequency content. We found significant correlations between the absolute error and these properties, with the exception of frequency. The absolute localization error was negatively correlated with a vocalizations energy (r = −0.13, p ≪ 0.001, Fig. 5G). Average frequency of the USV did not influence localization accuracy (r = −0.02, p = 0.27, Fig. 5H). Larger frequency ranges of USVs slightly improved localization accuracy (r = −0.06, p = 0.005, Fig. 5I). Lastly, longer durations of USVs also improved localization accuracy (r = −0.13, p ≪ 0.001, Fig. 5J). All correlations reported are Spearman rank correlations, as the functional relationships are clearly not linear. This pattern of results is expected since higher energy, larger frequency range and longer signals provide more information for the localization and may thus improve the signal-to-noise ratio. Conversely, a shift in absolute frequency would not be expected to influence localization performance.

### Sex-related differences in social vocalizations

We next investigated sex-specific differences in vocalizations during social interaction of CBA mice. The analysis was restricted to a limited set of basic properties (as above i.e. frequency, frequency range, duration, level) to demonstrate the use of attributing vocalizations to their emitters during social interaction. We find male and female USVs to differ significantly in duration, frequency, and to a lesser degree in frequency range and in level.

On average, male calls had a mean frequency of 73.5 kHz (S.D. = 5.65 kHz) during social interaction, while female calls displayed a mean frequency of 75.5 kHz (S.D. = 7.1 kHz). Mean frequency differed significantly between sexes (Wilcoxon rank sum test; p ≪ 0.001; Fig. 5J). The duration of male calls (51 ms, S.D. 20 ms) was also slightly but significantly longer than female calls (47 ms, S.D. = 19 ms, Wilcoxon rank sum test; p ≪ 0.001, Fig. 5H). The energy and frequency range only exhibited borderline significance (p = 0.026, Fig. 5G; p = 0.01, Fig. 5I respectively).

Differences in these properties were not reported previously to our knowledge for the present strain of mice^8, 40^. Potential explanations include strain differences (CBA/CaOlaHsd, current study, vs C57/BL6^8^ and wild california mice^40^) as well as experimental or recording conditions. Furthermore, due to the neutral experimental area used here, we expect calls emitted to relate mostly to courtship rather than territorial disputes (which can both occur in these contexts^8^), suggesting at least a partial difference in behavioural states across the aforementioned studies.

## Discussion

We have developed a precise sound localization method adapted to the study of multiple animals, for example during social interaction. Both artificial and natural sounds indicate that a precision of a few millimeters is achievable, even in the presence of acoustic reflections and scattering. When applied to interacting mice, our method allows the attribution of individual localizations to the interaction partners, enabling the study of social communication. We find differences in vocalization pitch, duration, frequency range, and energy between male and female vocalizations for CBA/CaOlaHsd mice.

### Areas of application for acoustic localization

The current method of USV localization can be used to study social interaction in mice and other vocal rodents. Application of this method is primarily useful for, but not limited to, behavioural^4, 6^ and genetic USV research^41, 42^. Other fields may also benefit from this method, for example if rodent social communication is used as a natural stimulus, such as multisensory integration and auditory research^35, 37^. In theory, localization can be done in any experimental paradigm enriching the collected data. Presently, our experimental approach limits localization to 1 dimension, but we propose a generalization to 2D below.

Reliable attribution of USVs to their emitter is of great benefit for behavioural USV research as it would allow a proper understanding of social vocalizations. USVs and their structure are strongly influenced by the context in which they occur (i.e. both a mouse’s internal and external environment^5, 7^), meaning that knowledge on the vocalizing animal is of key importance here. For instance, the structure of USVs during courtship can be studied in more detail, as recent studies conclude that indeed both male and female mice vocalize during this behaviour albeit to a different extent^33, 43^. However, little is known about the effect of sex on USVs. Application of this method will allow for more accurate study of male courtship USVs, and opens the door for studying female vocal behaviour during courtship.

Acoustic localization methods can also be used in the study of multisensory integration during social interactions^36, 37^. USVs can be utilised as natural stimuli in combination with whisker touch, visual input, or odor. Recent publications indicate that crossmodal representation is present in the auditory cortex^35^. Precise localization of the vocal source combined with automated whisker tracking^44^, can help establish a similar result for audio-tactile integration in the barrel cortex.

Finally, the use of an USV localization algorithm in order to attribute vocalizations to their emitter, reduces the necessity of devocalization, i.e. silencing the vocal cords by cutting the inferior laryngeal nerve^32^. This allows for a more natural interaction and therefore benefits the ecological validity of an experiment and improves animal welfare.

### Comparison with impulse response based sound localization methods

The precision at which a sound can be localised depends mainly on the acoustic properties of the environment (reverberations and scattering), combined with the analytical properties of the localization method. We have presented and compared a range of localization methods, which are largely agnostic about the acoustic properties, typically summarised as the impulse response of the environment. Hence, in principle, methods that incorporate this generative model of the recorded data should be advantageous. We have implemented and tested an existing method which directly incorporates the impulse response.

The impulse response generally depends only on the room, but for a given room, the positions of sound source and microphone are also relevant. Hence, in the present context of a moving sound source, the impulse response has to be reestimated for every vocalization. Mathematically, the problem takes the form

1 $${s}_{i}(t)={[s\ast {I}_{i}]}_{t}={\int }_{0}^{T}s(t-\tau ){I}_{i}(\tau )d\tau $$

where *s*_*i*_(*t*) denotes the recorded signal at microphone *i*, *I*_*i*_ the impulse response between source and microphone location, *s*(*t*) the sound at the source, and * denotes convolution. In order to estimate the inter-microphone delay, one can use the relationship

2 $${s}_{1}\ast {{I}_{1}}^{-1}=s={s}_{2}\ast {{I}_{2}}^{-1}$$

as previously proposed^45^. If the inversion of the two impulse responses is non-degenerate, the translation between the two microphone responses is given by

3 $${s}_{1}=({s}_{2}\ast {{I}_{2}}^{-1})\ast {I}_{1}={s}_{2}\ast {I}_{1,2}$$

Since convolution in the Fourier domain translates to a multiplication, the composite convolution kernel can be computed as

4 $${I}_{1,2}={F}^{-1}({S}_{2}/{S}_{1})$$

The maximum value of *I*_1,2_ can then be interpreted as the inter-microphone delay.

Alternatively, an iterative algorithm can be used to estimate *I*_1,2_, which, however, exhibited slow convergence on our dataset.

Using either the Fourier-based or iterative estimation, the estimates of this method remained noisy, and only led to interpretable values, under relatively ideal conditions (data not shown). The reason for this lack of precision is likely that the impulse response can differ substantially for each vocalization, depending on the relative position of the animals with respect to each other. A reason for this difference is the generally unstable deconvolution step, which may not have been constrained enough by individual vocalization. With sufficient data, this estimation generally works reliably, as e.g. in speaker calibration applied presently. For this reason, impulse response based methods are not presented here, although alternative estimation methods may prove more robust. The correlation based methods yielded overall substantially more robust estimates, despite being agnostic about the intervening impulse responses.

### Alternative correlation-based methods

In addition to the presented correlative methods, we tested two additional methods, one amplitude free, phase-based method, and one spectrogram based method. In the first method, the phases of each signal were computed on the basis of the Hilbert-transform. Next, cross-correlations were computed over very short time-intervals, e.g. 1 ms. All local maxima exceeding the median correlation were kept and combined into a histogram of local maxima. The histogram’s maximum in turn was taken as the inter-microphone delay. This method exhibited surprisingly precise estimates, exceeding all other methods, as long as the impulse response of the room was very brief (below the period of the vocalization, based on surrogate data). For the real data, the estimation quality was poorer than the above methods (data not shown).

The second, spectrogram based method resolves sound energy at different frequencies before estimating inter-microphone delays. The delay estimation is performed for a subset of frequency channels which are spanned by the sound, and the resulting correlograms are combined. Concretely, first a finely resolved short-term Fourier transform is computed for each channel, i.e. Fourier-transforms are computed over a sliding 4 ms (=1024 samples) window, which is moved sample-by-sample along the stimulus. Hence, one obtains a STFT at the same ‘resolution’ as the original sound, denoted as *S*_*i*_(*ωt*).

The combined cross-correlogram is computed as

5 $$C(\tau )=\sum _{\varpi \varepsilon {\rm{\Omega }}\,}\sum _{t=-\tau }^{T}Abs({S}_{1}(\varpi ,t))Abs({S}_{2}(\varpi ,t+\tau ))$$

where *Abs*(*S*) denotes the absolute value and Ω is the set of frequencies (chosen here to be the channels whose average activation is larger than the median activation across all channels). As before the best delay is chosen as $ma{x}_{\tau \varepsilon [-T,T]}(C(\tau ))$.

It provided a robust measure of location, especially for larger distances from the center, however, was overall inferior in estimation quality compared to the EWGCC. Further, its computation takes a factor 50 longer than all other methods, which poses some practical hurdles for large datasets.

### Generalization to localization in the plane

In the present experimental setup, animal motion was restricted to a single dimension in order to enable the local video recording during snout-snout interactions. While this type of setup has been used successfully for the study of rodent social interactions^36, 37^, the study of rodents in a plane or even 3D space is generally desirable, e.g. to study chasing behavior^33, 38^. A generalization of the present approach to 2D (or 3D) localization is possible, requiring a few additions on the practical and on the analytical level. Practically, a 2D arena needs to be equipped with at least 3 microphones. Analytically, the present approach needs to be generalised to the intersection of midlines between pairs of microphones, using Eq. 12 to correct for the distance away from the centerline connecting the microphones. Each midline corresponds to the points in the interaction plane consistent with the estimated relative position between a pair of microphones. For three microphones, the 3 midlines should intersect in one point, for 4 microphones there are 6 midlines, i.e. n choose 2 in general. Pilot experiments with 3 microphones suggest that the precision in a single dimension translates to each of the two dimensions, yielding an MAE of ~10–12 mm. Using more microphones, the certainty of the estimate could be further improved since these would define the intersection point more precisely, and outliers could be discarded. In addition, an open arena with sound proofed floor should be used to further reduce reflections and movement noises. While we do not foresee any major problems, the complete practical implementation may present some unexpected challenges, e.g. regarding computational implementation, runtime, and fusion of the individual estimates.

### Vocalization differences between sexes of CBA/CaOlaHsd mice and their anatomical correlates

**Figure 6:**
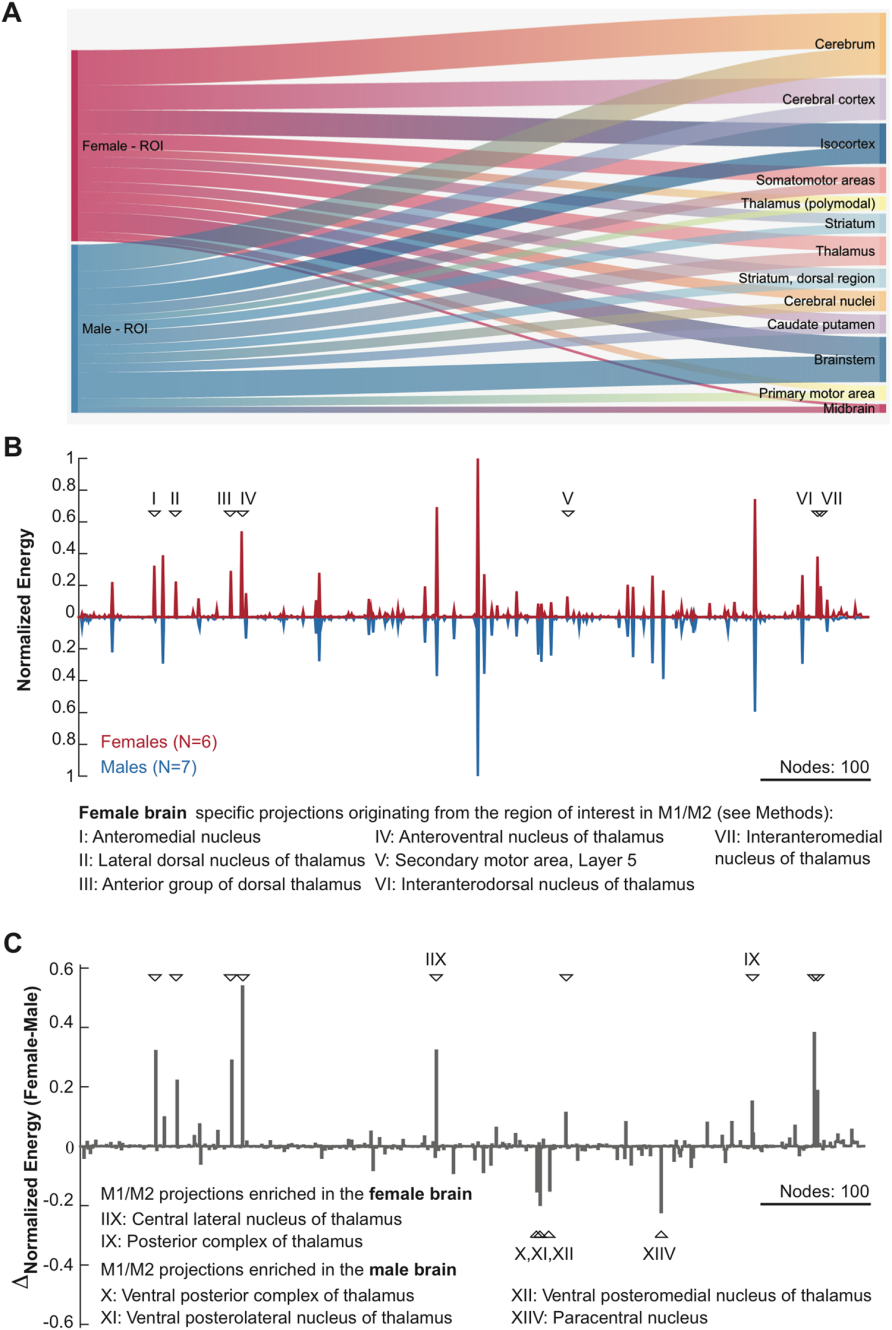
Sex differences in motor cortical projections in the mouse brain. Monosynaptic projections originating from a motor cortical region of interest (ROI) were quantified as described in the Materials and Methods section. (**A**) Major projection targets for the infragranular neurons in the ROI. The edge weight represents the relative weight of projections by volume. Only the top 13 targets are shown. (**B**) Normalised projection density across all nodes in the mouse brain. Downward triangles mark those nodes that receive monosynaptic input exclusively in the female brain. The numbers associated with triangle refer to the name of nuclei which are listed below the figure. (**C**) The difference of the normalised energy values across the sexes reveal the nodes that receive preferential input from either sex. Nodes marked with IIX and IX refer to descending nuclei that have preferential M1/M2 input in the female brain, compared to the male. Nodes, X-XIIV are nuclei that have preferential input in the male mouse brain.

While the present study focusses on improving the accuracy of localizing USVs, we also find a difference in the vocalization properties between male and female CBA/CaOlaHsd mice. Males emitted longer and lower frequency calls than females during the social interaction studied presently. To our knowledge this difference in vocalization properties has not been described before. Both of these differences are consistent with recent results from CBA/J mice (Fig. 6, although our results differ for call bandwidth)^6^, which, however, did not separate precisely between male and female animals. Generally, they are also in line with the male’s investment into courtship^46^, while putting themselves at a greater risk of discovery by a predator, for example due to the use of (slightly) longer vocalizations.

The neuronal basis of sex differences in vocalization is not known but likely to include vocal control by forebrain motor nuclei which has recently been investigated using transsynaptic tracers^47^. Sex-specific differences in these pathways are yet to be determined. To get a first insight into putative pathway differences, we performed a post-hoc analysis using a publically available data-set made available by the Allen Institute for Brain Science (http://connectivity.brain-map.org/, see Methods for details). We compared the monosynaptic projections originating from a spatially constrained region of the M1/M2, which was previously identified as a key structure for the generation of vocalizations in mice^47^. The results showed a higher prevalence of direct projections to the brainstem in males (Fig. 6A) and the differential projections via the cortico-truncal and cortico-thalamic descending pathways (Fig. 6B,C), including seven nuclei which receive forebrain motor input specifically in the female (see Fig. 6B for the list of the identified nuclei) as well as numerous thalamic nuclei that have preferential input in a sex-specific manner (Fig. 6C). These observations suggest that, in addition to differential vocal control by forebrain motor nuclei, potential sex-specific differences in auditory feedback during ultrasonic vocalizations, there might sex differences at the meso-scale circuit level which could contribute to sex-specific vocalizations. Future work, combining these circuit level differences with targeted, chronic recordings and local stimulation in the identified regions could shed light on the neural basis of sex differences in vocalization while providing a mechanistic insight into differential vocal control.

## Materials and Methods

All experimental procedures were approved by the animal welfare body of the Radboud University under the protocol DEC-2014-164 and conducted according to the Guidelines of National Institutes of Health.

### Animals

10 CBA/CaOlaHsd mice (6 male, 4 female) were studied in the experiments. The animals were 6 weeks old at arrival. The females arrived in two groups (2 animals each) of same age (possibly from the same litter), whereas the males arrived separated by two weeks, thus were all from different litters. The animals were housed socially but segregated by sex in individually ventilated cages. Animals had free access to food and water, and were kept on a 12 h/12 h light/dark cycle. After 2 weeks of acclimation the experiments were started.

### Experimental Procedures

Pairs of adult mice of different sexes were placed on two elevated platforms separated by a gap based on the gap-crossing task^48, 49^ (see Fig. 1). The experiments were conducted in an unlit box, except for an infrared backlight (see below) used in imaging. In the behavioural apparatus, animals performed without human interaction inside an automatised experimental setup. Prior to interaction, the platforms were initialised and positioned at a distance of 60 mm apart (before transferring the animals), thus allowing interaction, while effectively preventing them from crossing over. Platforms had a movable door, which opened at the beginning of the trial and closed at the end of it. The animals had time to interact for a period of 5–10 min, which was recorded using a broadband microphone (see below) and a high-speed camera (see below) for post-hoc analysis. Trial length was determined based on the activity of the mice and limited by computer memory to ~10 min for high speed video recording. Two sessions were collected daily per pair of animals. A total of 52 recordings of social interactions were analysed in the present study, comprising ~2200 vocalizations. This number may seem low in comparison with other studies, but during snout interactions are rarer than during free running interactions.

### Behavioural Apparatus

The behavioural apparatus was modified from the apparatus described in previous work^48^. In short, the apparatus consisted of two acrylic platforms (75 × 220 mm) surrounded by walls (height: 250 mm). Each platform was individually controlled by a stepper-motor driven, linear actuator, with a 0.005 mm resolution. Infrared emitter/sensor combination (IESC) were used for calibrating the starting position. Three other IESCs, positioned at the 9 mm, 78 mm and 183 mm from the front of the platform (10 mm above the platform floor), served to coarsely localize animals and trigger video acquisition (see below). For most experiments (40/52 experiments, 2006/2361 USVs) the platforms were additionally padded on the inside with acoustic foam (5 cm at the end, 1.5 cm at the sides, Basotect Plan50, BASF), to largely eliminate reflections inside the platforms. According to the manufacturer’s specifications, this should have removed all reflections above ~1 kHz.

Both motor commands and sensor information were provided via a Matlab-controlled DAQ card (PCIe-6353, National Instruments, Austin). Motor commands were transferred via an Arduino-circuit, powered by a regulatable power-supply (Voltcraft PPS-11603). Sensor read-outs and camera trigger times (see below) were directly acquired and digitised at 10 kHz. The apparatus was housed in a sound-proofed chamber, covered on the inside with acoustic foam (5 cm, as above).

### Video Recording and Animal Tracking

High speed video was recorded at 100–480 fps and digitised at 640 × 512 pixels (resolution of ~0.1 mm/pixel; Camera: PointGrey Flea3 FL3-U3-13Y3M-C, Monochrome, USB3.0). Part of the experiments was performed over a field of view of 70 × 57 mm (Lens: SainSonic XR-300, 35 mm, 1:1.7, 355 USVs), while the majority was collected for a bigger field of view of 164 × 205 mm (Lens: Cosmicar, 12.5 mm, 1:1.4, ~2000 USVs). The shutter time was set at 0.1 ms to minimize motion blur. Images were captured against a uniformly illuminated, rectangular infrared backlight (210 × 140 mm array of 1200 infrared LEDs (in 30 × 40 arrangement; *λ* = 850 nm), with a glass diffuser in front. As such, mice appear black against a bright background. Video acquisition was triggered when both animals were located at the gap, and terminated, when an animal left the center. Images were transferred to memory and stored for analysis. Offline, mice were tracked in the XY-plane by a human observer (J.J.H.) at the midpoint of every vocalization using a custom, Matlab-based visualization tool. The observer marked the snout and the point between the ears, generating a viewing direction vector for each mouse. The reference position for comparison with the acoustic location was at 10% of the distance from the snout towards the ears.

### Audio Recording

Sounds inside the booth were recorded with two ultrasonic microphones (CM16/CMPA48AAF-5V, flat (+/−5 dB) frequency response within 7–150 kHz, AviSoft, Berlin) at a sampling rate of 250 kHz. An analog low-pass filter at 120 kHz prevented aliasing and excluded contributions beyond 120 kHz. Recorded data was digitised using a second DAQ card. The microphones were placed centered on the midline connecting the platforms, at a symmetric distance of 230 mm from the center of the gap between the platforms at a height of 354 mm above the platform floor. The entrance of the microphones was aimed at a 45° angle with respect to the horizon towards the gap. Based on the microphone’s angle of receptivity (~25 dB attenuation at 45°), the microphones receive sounds from both platforms. To account for sensitivity differences, the responses of the two microphones were scaled by their average response to Gaussian white noise (relative correction factor = 0.74, confirmed by the manufacturer to be within tolerances).

### Detection of Ultrasonic Vocalizations

Mouse USVs were detected automatically using a set of algorithms developed by Holy and Guo (2005). Briefly, these algorithms compute a short-term Fourier transform with half-window overlaps (window length ~1 ms). The spectrogram is then thresholded and median filtered to remove isolated, low-amplitude speckles. The spectral purity was computed for each time-slice by dividing the maximal power in a bin by the summed power in this time-slice. Sounds were accepted if their average frequency was >25 kHz and their spectral purity was >0.1. Vocalization discovered on one microphone, were considered to be present on both microphones, and the corresponding data was kept for both sides. Based on visual inspection, the algorithm reliably detected USVs, if their signal to noise ratio exceeded ~0.5 (on the sound pressure level). Properties of individual USVs were extracted on the basis of their spectrogram, i.e. amplitude, average frequency, frequency range and duration (see Supplementary Figure 2).

### Localization of Ultrasonic Vocalizations

The symmetric arrangement of the microphones allowed spatial localization of sounds in one dimension, i.e. along the line connecting the microphones. Both temporal and level differences of arrival at the microphones can be utilised for this purpose. Temporal differences of arrival allow a difference of 4 µs (1 sample at 250 kHz) to be resolved between both sides. Based on the speed of sound in air this translates to an upper bound of the localization accuracy of ~1.37 mm. Differences in level are not as precise, but can be used to resolve certain ambiguities, e.g. if animals are very close in space, the vocalization can be attributed to the animal facing the microphone where the level was greater (see below).

We compared the performance of three temporal localization techniques, basic cross-correlation (CC), generalised cross-correlation (GCC) and a novel extension, envelope weighted generalised cross-correlation (EWGCC). Importantly, these techniques only yield an estimate of the inter-microphone delay, which translates into different spatial positions depending on the relative vertical position of the microphones compared to the sound source. Since we could not find a treatment in the literature of this conversion, it is provided below in detail.

### Conversion of Delay to Position

If a sound is emitted between two microphones, the arrival time at the microphones is determined by the distance of the sound source to each microphone. In the present setup this distance has a horizontal and a vertical component. Given the position of the microphones and sound source, the inter-microphone delay *ΔT* can simply be computed by the product of the different in path-lengths *ΔP* divided by the speed of sound ${v}_{sound}$ (see Fig. 2A for a visualization of the variables). *ΔP* can be computed by the application of the Pythagorean theorem, which comes out to be

6 $$\Delta P(D,H,\Delta X)=(\,\sqrt{\,{(\frac{D}{2}+\Delta X)}^{2}+{H}^{2}}-\sqrt{{(\frac{D}{2}-\Delta X)}^{2}+{H}^{2}}\,)\,$$

where *H* is the vertical distance between the sound source and the center of the microphone membrane, *D *is the horizontal distance between the two microphones and *ΔX* the horizontal distance of the sound source from the center between the two microphones (see Fig. 2A for a visualization of the variables).

The inverse problem, i.e. estimating the sound position from the inter-microphone delay is more complicated. We start from the above forward equation, and square both sides:

7 $$\Delta {P}^{2}={(\frac{D}{2}+\Delta X)}^{2}+{(\frac{D}{2}-\Delta X)}^{2}+2{H}^{2}-2\sqrt{{(\frac{D}{2}+\Delta X)}^{2}+{H}^{2}}\sqrt{{(\frac{D}{2}-\Delta X)}^{3}+{H}^{2}}.$$

Rearranging and squaring again yields

8 $$4\,({(\frac{D}{2}+\Delta X)}^{2}+{H}^{2})\,({(\frac{D}{2}-\Delta X)}^{2}+{H}^{2})={({(\frac{D}{2}+\Delta X)}^{2}+{(\frac{D}{2}-\Delta X)}^{2}+2{H}^{2}-\Delta {P}^{2})}^{2}.$$

After a few steps of expansion and cancellation, we arrive at

9 $$4\,(\Delta {X}^{4}+(2{H}^{2}-\frac{{D}^{2}}{2})\Delta {X}^{2}+({H}^{2}+\frac{{D}^{2}}{4}))=4\Delta {X}^{4}+4(\frac{{D}^{2}}{2}+2{H}^{2}-\Delta {P}^{2})\Delta {X}^{2}+{(\frac{{D}^{2}}{2}+2{H}^{2}-\Delta {P}^{2})}^{2}$$

and simplifying and canceling terms further yields

10 $$-2{D}^{2}\Delta {X}^{2}+{(2{H}^{2}+\frac{{D}^{2}}{2})}^{2}=(2{D}^{2}-4\Delta {P}^{2})\Delta {X}^{2}+{(2{H}^{2}+\frac{{D}^{2}}{2}-\Delta {P}^{2})}^{2}.$$

Collecting terms for the sought variable *ΔX*, and using the binomial relationship ${a}^{2}-{(a-b)}^{2}=(2a-b)\,b$ with $a=2{H}^{2}+\frac{1}{2}{D}^{2}$ and $b=\Delta {P}^{2}\,\,$gives

11 $$4({D}^{2}-\Delta {P}^{2})\Delta {X}^{2}=\Delta {P}^{2}(4{H}^{2}+{D}^{2}-\Delta {P}^{2}),$$

which yields the final conversion formula, after solving for *ΔX*:

12 $$\Delta X(D,H,\Delta P)=\frac{1}{2}\Delta P\sqrt{\frac{4{H}^{2}+{D}^{2}-\Delta {P}^{2}}{{D}^{2}-\Delta {P}^{2}}}\,$$

Hence, we have derived an explicit formula for the horizontal position *ΔX* as the function of path-length difference *ΔP* or the inter-microphone delay *ΔT* via ${v}_{sound}$, given the microphone height *H* and distance *D*.

Next, we describe the three estimation methods. Prior to application of each technique, vocalizations were automatically detected and separated, using extensions of the work by Holy & Guo^4^, as well as high-pass filtered signal with a corner frequency of 10 kHz (4th order, Butterworth-filter), to prevent environmental noise from entering the estimate. All localization measures will be made available as a toolbox on our lab website.

### Cross correlation measures

Ideally, the signals at the two microphones are just time-shifted versions of each other. In classical cross-correlation (referred to as simple cross correlation hereafter) its maximum across all possible delays *τ* is the most obvious choice for the difference in travel time to the microphones. For the two microphone recordings *s*_1_(*t*) and *s*_2_(*t*), it is defined as

13 $$\Delta T=ma{x}_{\tau \varepsilon [-T,T]}\sum _{t=-T}^{T}{s}_{1}(t){s}_{2}(t+\tau )$$

where *Δ*T is an arbitrary maximal delay, which in the present case should be chosen to cover all possible delays for the given environment, i.e. at least corresponding to the distance between the microphones. Cross-correlation estimates are provided in Fig. 3 (black).

### Generalised Cross-Correlation

Several non-idealities in the acoustics of the environment may introduce additional transformations in the signals, in which case the solution provided by simple cross correlation may not always correspond to the actual time delay. In the present case, these transformations are mostly acoustic reflections from the apparatus, slight differences between the spectral properties of the microphones and the typically harmonic nature of the vocalizations. The generalised cross correlation was computed as

14 $$G(\varpi )=\frac{{S}_{1}{(\varpi )}^{\ast }}{|{S}_{1}(\varpi ){S}_{2}{(\varpi )}^{\ast }|}$$

where ${S}_{i}(\omega )$ denote the Fourier transform of the microphone signals, and ${S}^{\ast }$ the complex conjugate of a signal. The denominator here serves to equalize the weight across frequencies, which can make use of phase-differences in quieter frequency channels. The final inter-microphone delay was then obtained by computing the inverse Fourier transform of G, and extracting the timing of the peak (as in Eq. 13).

### Envelope Weighted Generalised Cross-Correlation

The normalization step in the generalised cross-correlation (Eq. 14, denominator) is often helpful, but can also have detrimental effects on the localization quality if frequency channels contribute that contain only noise. We accounted for this problem by computing the envelope across frequencies, and only including frequency channels whose amplitude was >5% of the peak channel. The envelope was computed empirically as the average over 10 consecutive frequency bins, and then interpolated to the full set of frequencies again. This modification improved the robustness of the GCC especially for rather narrow-band USVs.

### Localization Quality Criterion

The quality of single localization estimates can vary across different vocalizations, most likely due to other environmental noises. If the quality was known for individual vocalizations, the assignment could be selectively performed for localizations of different quality, e.g. in relation to the minimal distance to the animals. We attempted to estimate the quality of a single vocalization by computing the signal-to-noise of the peak in the final time-resolved correlation (from either of the three methods) compared to the mean correlation across the entire range of delays. We term this measure a *correlation quality measure* (CQM), and results are reported as a function of the quality criterion in Figs 4 and 5. We find that higher CQM values lead to lower localization errors, confirming that the CQM is informative on a single USV level.

### Disambiguation during Close Range Interactions

When the mice interact closely, the position of the snouts can fully overlap, thus creating an ambiguity that cannot be resolved based on 1D localization. In order to disambiguate the situations, we additionally used the relative level at the two speakers to determine the emitting mouse. Concretely, if the animal snouts were within 2 times the MAE for 1D localization (Fig. 4), i.e. 2 × ~5 mm = ~10 mm, we attributed the vocalization to the animal facing this speaker, i.e. on the opposite platform. We verified the validity of this approach by applying the same criterion to the single mouse vocalizing in different directions and comparing the resulting levels. For the head orientations in snout-snout interaction, the method showed an average accuracy of 88% (see Fig. S3 for more details). Vocalizations outside of this close range were attributed based on position alone.

### Verification of Localization Performance

The localization performance of the individual algorithms was first tested using artificially generated sounds, and further with a single vocalizing male mouse. For this first test, a high-fidelity speaker (Fostex T250D) was digitally calibrated to produce equal output level within the relevant range (10–100 kHz, see Fig. S1A). Briefly, the calibration was performed by placing the speaker at a distance of 50 mm in front of the microphone. First, a Gaussian white noise was played, to estimate the frequency transfer function of the speaker (function *tfestimate* in Matlab). This function was then divided by the intended flat spectrum, to arrive at the inverse transfer function. The inverse impulse response (IIR) was computed via the inverse Fourier transform of the inverse transfer function. Subsequently, stimuli were convolved with the IIR to achieve an equalised output. The time-delay introduced by the product of the IIR and the speakers impulse response (3 ms) was applied as a shift to the stimulus, when comparing input and output. The equalization spectrum and amplitude were tested by presenting white noise as well as Chevron-vocalization shaped sounds, i.e. an up-flat-down frequency sweep (see Fig. S1B,C).

For testing the localization algorithms, the membrane of the speaker was positioned at the same height as the mouse heads durings interaction (i.e. ~1 cm above the platform) at a number of horizontal positions (−50 mm to 50 mm relative to the center, in steps of 5 mm). This range covered more than the visible range of the camera (±28 mm). Localization performance was evaluated for the white noise (see Fig. 3B).

In addition to the testing based on synthetic stimuli, the quality of this estimate was assessed by estimating the sound source location of real USVs emitted by individual male mice (n = 3). For this experiment a single male mouse was placed on an elevated, elongated platform (400 mm × 100 mm, at 250 mm above the floor), flanked by two microphones (see Fig. 4A for the spatial arrangement, locations ±296 mm, at a height of 133 mm relative to the platform level). In this test, we chose to sample a larger range of distances than used in the gap interaction task, in order to explore also locations close to the microphones. A digital camera (Point Grey, Flea3) was placed centered over the platform (height relative to platform level: 850 mm) to obtain the mouse position as a function of time. Recordings were performed at 100 Hz at a resolution of 640 × 512 pixels over a viewfield of 420 × 336 mm (Lens: Cosmicar, 12.5 mm, 1:1.4). The setup was surrounded by acoustic foam (5 cm, as above) to prevent inside reflections and outside noises from entering. In order to motivate the male mouse to vocalize a female mouse was briefly presented repeatedly for snout-snout interaction (when the male mouse had ceased to vocalize for ~30 s) and then removed to a sound-proofed box. Results from this analysis are shown in Fig. 4B–D.

### Projection mapping

To address whether observed sex differences correlate with motor cortical projections we studied the anatomical projections originating from a region of interest (ROI) across sexes using the data made available by the Allen Brain Institute. The, so called, Mouse Connectivity database (http://connectivity.brain-map.org) currently includes 2546 experiments where a select population of neurons express a fluorescent protein as well as the whole brain visualization of the projections of the fluorescently stained neurons, thus providing a database to study connectivity in the mouse brain in the mesoscale.

We have chosen the ROI and cortical layers of interests relevant to sex differences in motor cortical projections based on the transsynaptic mapping experiments by previous work^47^ (see e.g. Fig. 3) who showed that infragranular layers in a region of primary (M1) and secondary (M2) cortices contribute to control of ultrasonic vocalizations. Spatially cross referencing their transsynaptic tracer visualization and egr-1 based activity mapping results with the reference mouse brain atlas of the Allen Brain Atlas, we selected 13 experiments (6 female, 7 male) which were performed in transgenic mouse strains and allowed mapping of projections originating from the infragranular layers within the ROI. The strains used were: Tlx3-Cre_PL56, Syt6-Cre_KI148 and Sim1-Cre_KJ18. Unique experiment identifiers are: Female – 177319974, 168229113, 297947641, 287807743, 297892130, 294525944. Male – 122642490, 156786234, 297854981, 297711339, 297714071, 293432575, 293431869. All data are available from the Allen Institute for Brain Science at http://connectivity.brain-map.org/.

To determine the main projection targets of the ROI (see Fig. 6A), we calculated the normalised (to the maximum) projection volume. To determine the relative “strength” of projections, we calculated the normalised (to the maximum) projection energy where projection energy is defined as the total intensity of tracer signal/total number of pixels in a given node. Thus projection energy can be used to infer the relative “strength” of projections into a given node in the network.

### Statistical Analysis

To avoid distributional assumptions all statistical tests were nonparametric, i.e. Wilcoxon rank sum test for two group comparisons, Kruskal-Wallis for single factor analysis of variance. Correlation is computed as Spearman’s rank based correlation coefficient. Error bars represent standard errors of the mean (SEM), unless stated otherwise. All statistical analyses were performed in Matlab (The Mathworks, Natick) using functions from the Statistics Toolbox.

### Data Availability Statement

The authors declare that they will make the original data readily available to anyone upon request.

## Electronic supplementary material

Supplementary Figures and Captions
