# Supplementary material for: High-precision spatial localization of mouse vocalizations during social interaction
Source: Sci Rep. 2017 Jun 7;7:3017. doi: 10.1038/s41598-017-02954-z (PMC5462771; doi:10.1038/s41598-017-02954-z)
Supplement: Supplementary file 1 — Supplementary Figures and Captions [file 41598_2017_2954_MOESM1_ESM.pdf]

Supplementary Information

for

High-precision spatial localization of mouse vocalizations during social interaction

Jesse J. Heckman[1], Rémi Proville[1], Gert J. Heckman[2], Alireza Azarfar[1], Tansu Celikel[1], Bernhard Englitz[1]

Supplementary Figures:

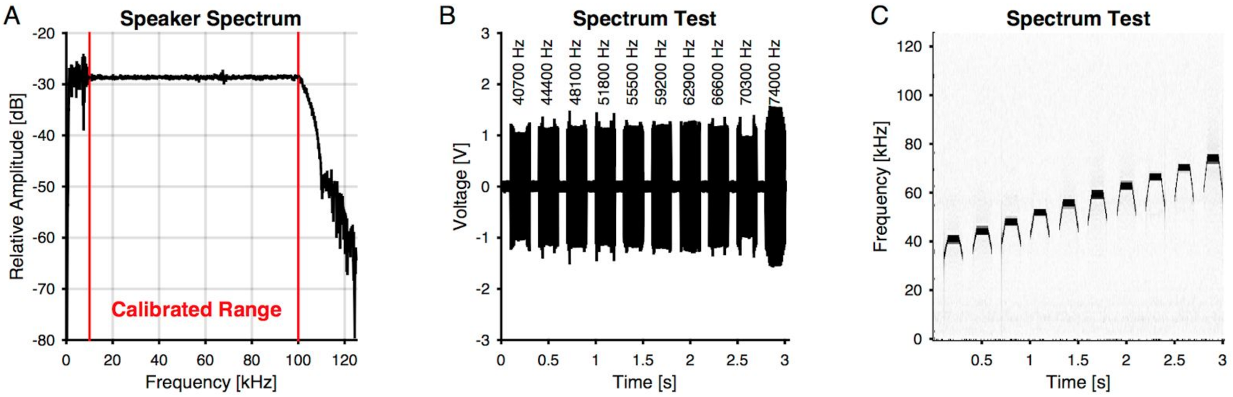

Figure S1: Spectral properties of the test speaker.

(A) A high-fidelity speaker (Fostex T250D) was digitally calibrated to produce equal output level within the range of 10-100kHz (red range). For calibration, the speaker was positioned at a defined distance directly facing the microphone, and a white noise voltage stimulus was presented, recorded and digitized. The Fourier amplitudes (black) of the recorded sound (after calibration) show only variations within a few decibels around the equalization level. For details on the calibration see Methods.

(B,C) The equalization across level was additionally verified using a sequence of sounds, mimicking Chevron-vocalizations of ascending frequency. The recorded sound **(B)** and its spectrogram **(C)** show only small variations in amplitude, with some residual variations at the beginning and end of the sound, where the frequency varies rapidly

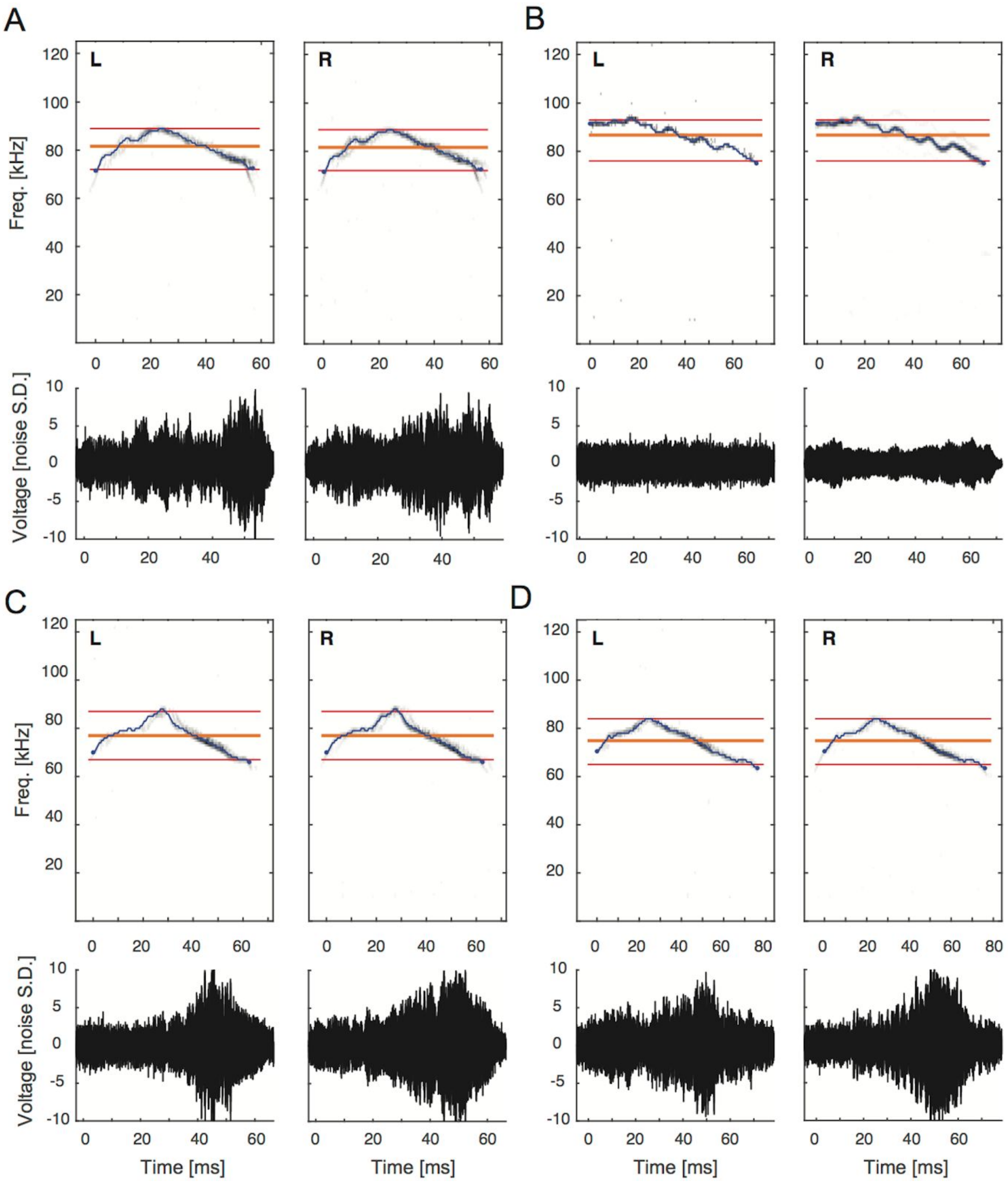

Figure S2: Examples of extracted vocalizations.

A-D Four examples of vocalizations extracted automatically on the basis of their spectral purity, amplitude, and temporal contiguity. For each vocalization, a range of properties was extracted, including the average frequency, frequency range, duration, amplitude, number of components, etc, indicated in the figure.

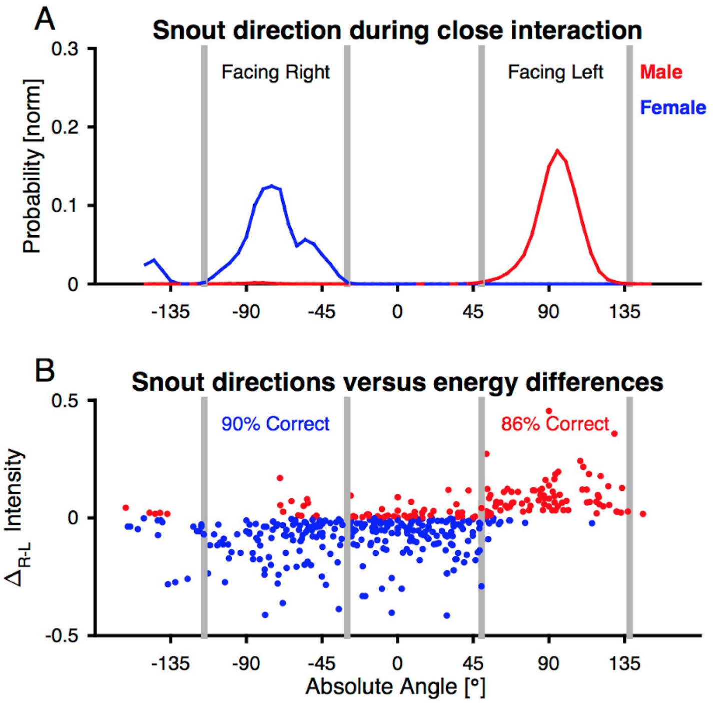

Figure S3: Ground truth for assignment for intensity difference assignment during close interaction

A During the facial interaction paradigm over the gap, male and female mice were typically in close contact, however, facing in opposite directions. Male mice (red), placed on the left platform faced to the right (90 to 100º), with angles quite constrained between 45 and 135 degrees. Female mice (blue), placed on the right platform, faced mainly to the left (-76 to -90º), covering dominantly a range of -40º to -110º, with a few outlier at lower degrees. Since the present analysis investigates vocalization during close interaction, only snout-snout distances of <10mm are included here.

B During close interaction, we assigned the emitting animal on the basis of the relative intensity at the two microphones. Hence, we checked the relative intensity of vocalizations at the microphones for the single male mouse, since here the emitter is known. Within the angle ranges during interaction (compare to **A,** demarcated by gray lines), intensity difference was a reliable predictor for angle of emission, with 90% correct for leftward, and 86% correct for rightward facing mice. Correspondingly, during snout-snout interaction, the correct animal would thus be identified by the intensity difference.
